# Supplementary figures and images for: Enhancement of a protocol purifying T1 lipase through molecular approach
Source: PeerJ. 2018 Nov 16;6:e5833. doi: 10.7717/peerj.5833 (PMC6241395; doi:10.7717/peerj.5833)

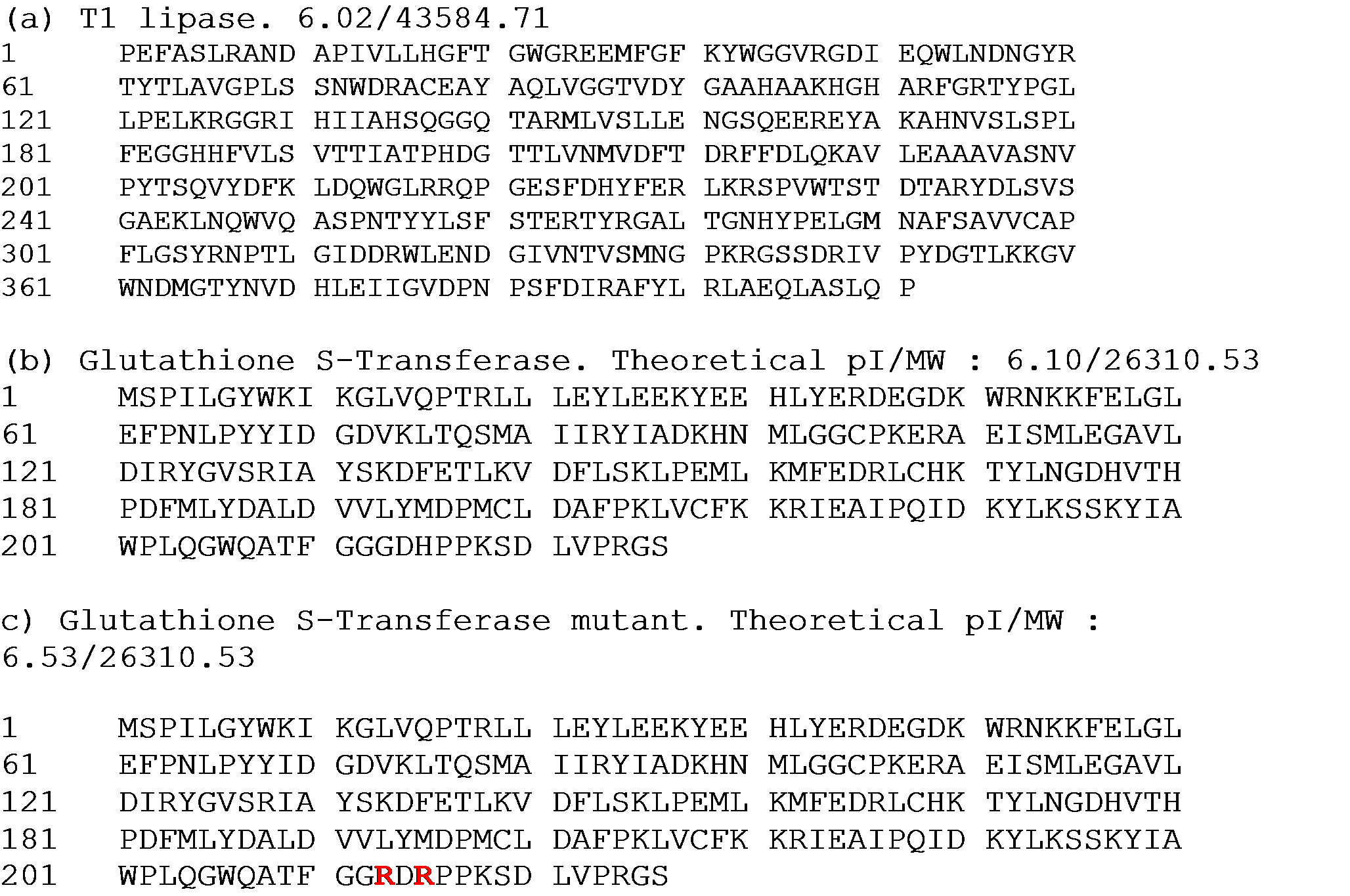

Supplement: Supplemental Information 1 — (a) Theoretical pI value of T1 lipase. (b) Theoretical pI value of GST tag. (c) Theoretical pI value of GST tag after replacing two points of GST sequences. Glycine at position 213 replaced with Arginine (G213R) and Histidine at position 215 replaced with Arginine (H215R). [file peerj-06-5833-s001.png]

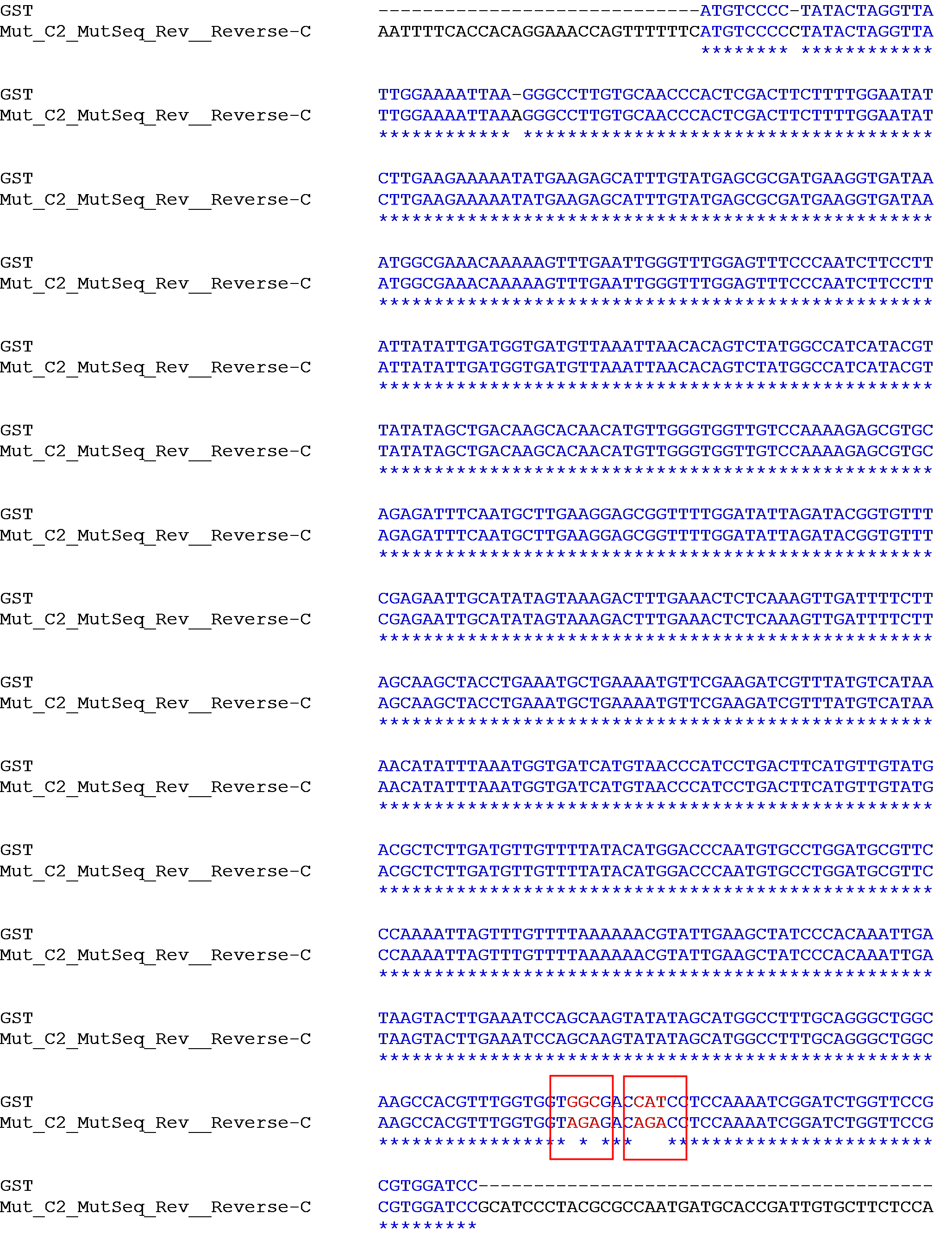

Supplement: Supplemental Information 2 — Two points of mutation were successfully created at position 213-GGC (Glycine) changed to AGA (Arginine) and position 215-CAT (Histidine) changed to AGA (Arginine). [file peerj-06-5833-s002.png]

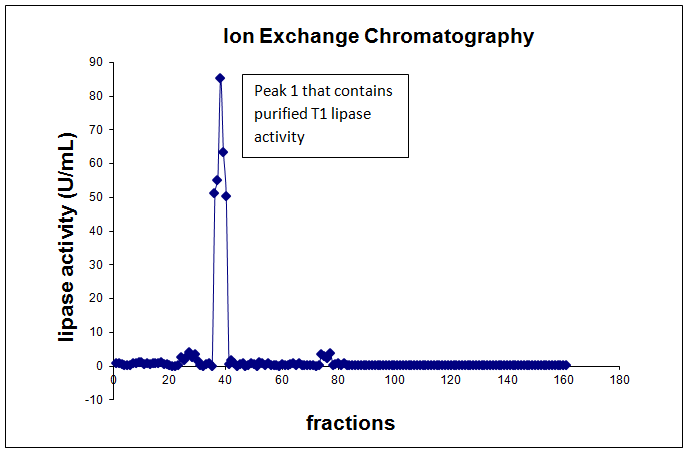

Supplement: Supplemental Information 3 [file peerj-06-5833-s003.png]
